# The Effects of Age, Period, and Cohort on Mortality from Ischemic Heart Disease in China

**DOI:** 10.3390/ijerph14010050

**Published:** 2017-01-07

**Authors:** Jie Chang, Boyang Li, Jingjing Li, Yang Sun

**Affiliations:** 1 Department of Pharmacy Administration and Clinical Pharmacy, School of Pharmacy, Xi’an Jiaotong University, Xi’an 710061, China; jiechang@xjtu.edu.cn; 2 Center for Drug Safety and Policy Research, Xi’an Jiaotong University, Xi’an 710061, China; 3 School of Medicine and Health Management, Tongji Medical College, Huazhong University of Science and Technology, Wuhan 430030, China; jimmylee1900@gmail.com; 4 Department of Behavior Science and Health Education, Rollins School of Public Health, Emory University, Atlanta, GA 30322, USA; jingjing.li@emory.edu; 5 Department of Epidemiology, School of Public Health, Wuhan University, Wuhan 430071, China; 6 Department of Public Affairs and Management, School of Political Science and Public Administration, Wuhan University, 299 Bayi Road, Wuchang District, Wuhan 430072, China

**Keywords:** age-period-cohort analysis, ischemic heart disease, mortality, China

## Abstract

In contrast with most developed countries, mortality due to ischemic heart disease (IHD) continues to rise in China. We examined the effects of age, period, and cohort on IHD mortality in urban and rural populations from 1987 to 2013 to identify the drivers of this trend. Region-specific data on annual IHD mortality among adults aged 20 to 84 years and corresponding population statistics were collected. We then tested for age, period, and cohort effects using the Intrinsic Estimator approach. Our results indicated that IHD mortality in China increased significantly over the three decades studied. There was a log-linear increase in the age effect on IHD mortality as those aged 80–84 showed 277 and 161 times greater IHD mortality risk than those aged 20–24 in urban and rural populations, respectively. While there was an upward trend in the period effect in both populations, the influence of the cohort effect on mortality decreased over time for those born from 1904 to 1993. The age, period, and cohort effects on mortality in China were generally comparable between urban and rural populations. The results suggest that population aging is a major driver behind the rapid rise in IHD mortality. Increased exposure to air pollution may also have played a role in driving the period effect

## 1. Introduction

Ischemic heart disease (IHD) is a leading cause of morbidity and mortality worldwide [1,2,3] and is responsible for over 7 million deaths annually—or 13% of all-cause mortality [4,5]. Although a rising trend in IHD mortality rates has been observed since the beginning of the 20th century, this trend was found to have reversed in most industrialized countries after IHD mortality rates reached a peak by the 1970s owing to improvements in both treatment and prevention [1,6,7]. By contrast, mortality due to IHD continues to rise in many low- and middle- income countries [8,9,10,11].

Variation in mortality rates over time is strongly attributable to influences that vary by age, calendar period of death, and birth cohort [12]. Among these, the cohort effect has received relatively little attention [13]. However, the influence of different risk factors for IHD mortality can vary with both the age of exposed individuals and level of exposure over time [14]. Analyses based on the assumption that changes in mortality rates over time are equal across birth cohorts, depend on rates of change in period-specific risk exposure, or are independent of an individual’s year of birth, may lead to unreliable or even misleading conclusions. It is therefore necessary to analyze the influences of age, period, and cohort effects together to fully understand the drivers behind trends in IHD mortality over time.

China has experienced rapid economic growth and social change since the adoption of economic reforms from 1978 onwards, which has in turn resulted in substantial changes in lifestyle for the majority of the population. Like many other developing countries, China is confronting a double burden of disease as a result of the epidemiologic transition. Non-communicable diseases represent a major challenge, both adversely affecting population health and increasing the financial burden on China’s healthcare system [15]. Based on findings of the Global Burden of Disease Study 2015, the number of deaths attributable to IHD increased from 7648.4 thousand in 2005 to 8917.0 thousand 2015 [16]. Furthermore, IHD represented the second leading cause of years of life lost among the Chinese population in 2015 [16].

Age-period-cohort (APC) analysis can identify net age, period, and cohort effects from age-specific mortality data gathered over time. It has played a critical role in identifying risk factors underlying mortality from different causes in a macroscopic context. While previous analyses have estimated age, period, and cohort effects on IHD mortality in other countries and regions [17,18,19,20,21,22], Chinese data have been used to estimate the impact of population aging on coronary heart disease on the national level [23], predict future trends in prevalence and incidence of cardiovascular disease [24], and analyze the upward trend in coronary heart disease in Beijing City [25].

We sought to quantify the age, period, and cohort effects on secular trends in IHD mortality in urban and rural China by using national statistics over an extended period to inform long-term national IHD prevention strategies.

## 2. Methods

### 2.1. Data Sources

IHD cases were defined using the International Classification of Diseases (ICD) codes ICD-9 410.00–414.9 for data collected prior to 2002 and ICD-10 I20–I25 for data from 2002 onwards. Official national-level data on annual IHD mortality rates were extracted from the Chinese Health Statistical Annual Report (1987–2001) and the Chinese Health Statistics Yearbook (2003–2013). The mortality statistics obtained were based on events recorded by the Ministry of Health-Vital Registration (MOH-VR) System, which has been shown to provide a high degree of reliability. Corresponding information about urban and rural population by five-year age groups were derived from the third to the sixth National Population Census (1982, 1990, 2000 and 2010). Using these two data sources, we calculated age- and region-specific IHD mortality among those aged 20–84 for each five-year period between 1987 and 2013.

Data gathered from 1987 to 2013 were included in the full APC analysis and were tabulated to yield 13 five-year age groups from 20–24 to 80–84 years, six quinquennial time periods between 1987 and 2013 (1988, 1993, 1998, 2003, 2008, 2013), and 18 consecutive cohorts including those born in 1904–1908 and aged 80–84 years in 1988 to those born in 1989–1993 and aged 20–24 in 2013.

While data from individuals aged 0–19 was not analyzed owing to the extremely low incidence of IHD in this group, data from those aged 85 years and older were not included as this was an open-ended age group and mortality data were not available by five-year age brackets.

### 2.2. Statistical Analysis

Estimates of mortality rates were fitted using a basic APC model, which was initially operationalized as a linear regression equation:

*M_ij_* = *D_ij_/P_ij_* = μ + α*_i_* + β*_j_* + γ*_k_* + ε*_ij_* (1)

where *M_ij_* denotes the observed death rate for the *i*th age group for *i* = 1,…, *a* age groups in the *j*th time period for *j* = 1,…, *p* time periods of observed data, *D_ij_* denotes the number of deaths in the *ij*th group, and *P_ij_* denotes the estimated population size of the *ij*th group. Furthermore, μ denotes the model intercept or adjusted mean, *α_i_* denotes the *i*th row age effect or the coefficient for the *i*th age group, *β_j_* denotes the *j*th column period effect or the coefficient for the *j*th time period, γ*_k_* denotes the coefficient for the *k*th cohort for *k* = 1,…, (*a + p* − 1) cohorts (with *k* = *a* − *i + j*), and ε*_ij_* denotes the random error term with the expectation that E(ε*_ij_*) = 0.

Each of the three time-dependent variables is a function of the other two, such that the independent effects are difficult to separate. This results in the “identification problem”, for which a number of solutions exist—each with their own set of limitations [26,27]. We therefore applied the intrinsic estimator (IE) method [28], which has recently been used in a similar context to study secular trends in disease incidence and cause-specific mortality [29] and is considered to achieve model identification with the fewest assumptions.

Statistical analyses were conducted using Stata 12.0 (StataCorp LP, College Station, TX, USA) using the “apc_ie” command. The outcome variable, IHD mortality, was log transformed and odds ratios were calculated for each model parameter to allow comparison of mortality risk. We fitted three one-factor models, two two-factor models, and a full three-factor model to analyze each possible combination of age, period, and cohort effects. The Akaike information criterion (AIC) and the Bayesian information criterion (BIC) were then used to evaluate goodness-of-fit for each model.

In addition, we conducted a sensitivity analysis based on data adjusted for the change in the ICD to examine whether the change from ICD-9 to ICD-10 has caused notable impact on our results and conclusions. We first quantified the impact of the ICD change by deriving adjusting factors for urban and rural populations (dividing the overall IHD morality rates of urban and rural population of 2003 by the rates of 2001, respectively). Then we deflated all the data collected prior to 2002 based on ICD-9 using the adjusting factors to generate an adjusted data set for modeling. The results of APC analysis with IE approach using the adjusted data are shown in the Supplementary Materials. The approach of adjustment has been used in previous APC studies for similar purpose [30].

## 3. Results

### 3.1. Descriptive Analysis

**Figure 1:**
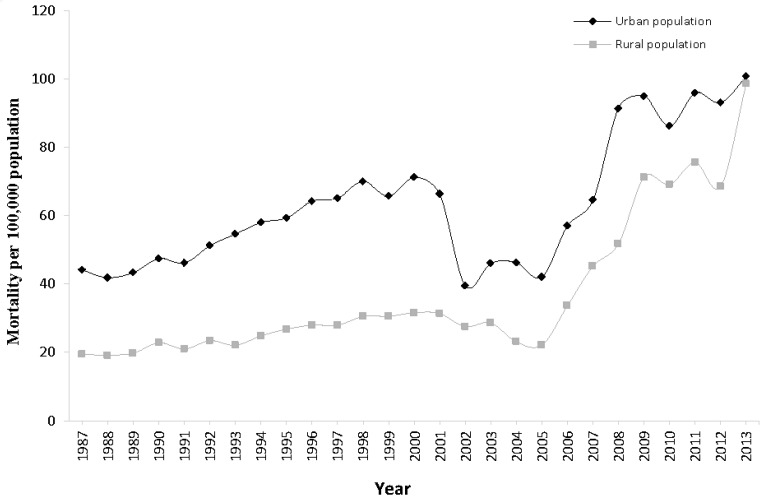
Ischemic heart disease (IHD) mortality rates trend in urban and rural China, 1987–2013.

Figure 1 shows the overall secular trends in IHD mortality in urban and rural populations from 1987 to 2013. Mortality increased by approximately 2.5 and 5 times in urban and rural populations respectively over this period. Despite fluctuations, IHD mortality rates in urban areas were consistently higher than in rural rates in these years—although the gap narrowed towards the end of the period as mortality in the rural population increased rapidly from 2005 and converged with that in urban areas by 2013.

**Figure 2:**
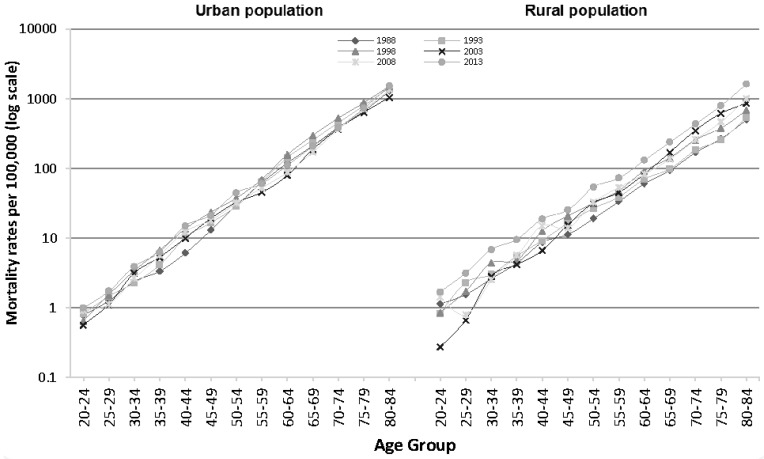
Age-specific IHD mortality in urban and rural China, by time period.

Age-specific IHD mortality rates by period are shown in Figure 2 for urban and rural populations. Overall IHD mortality rates increased with age following a log-linear trend such that IHD mortality risk among those aged 80–84 and 20–24 differed almost 1000-fold in both populations.

**Figure 3:**
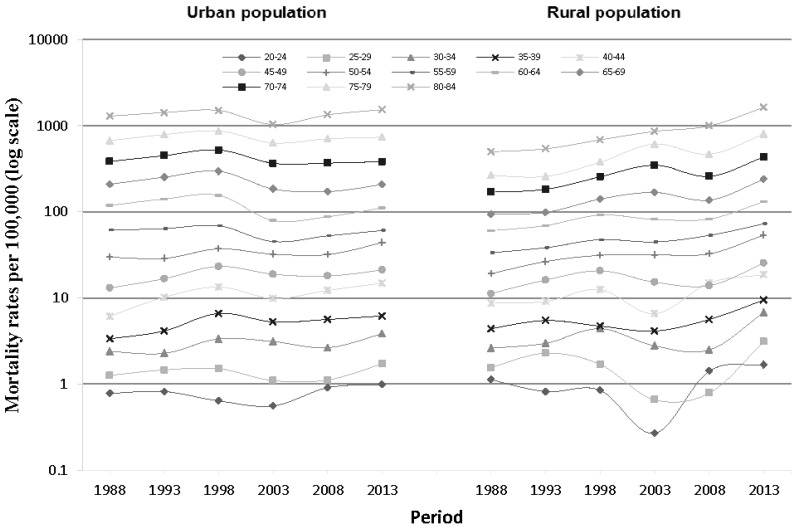
Period-specific IHD mortality rates in urban and rural China, by age group.

Period-specific IHD mortality rates by age group in urban and rural populations are shown in Figure 3. While mortality increased consistently among those aged 50–54 and 80–84 in rural areas, all other groups experienced fluctuating mortality risk over time without a discernible trend. Mortality risk was least stable among those aged less than 45 years—particularly in rural areas.

**Figure 4:**
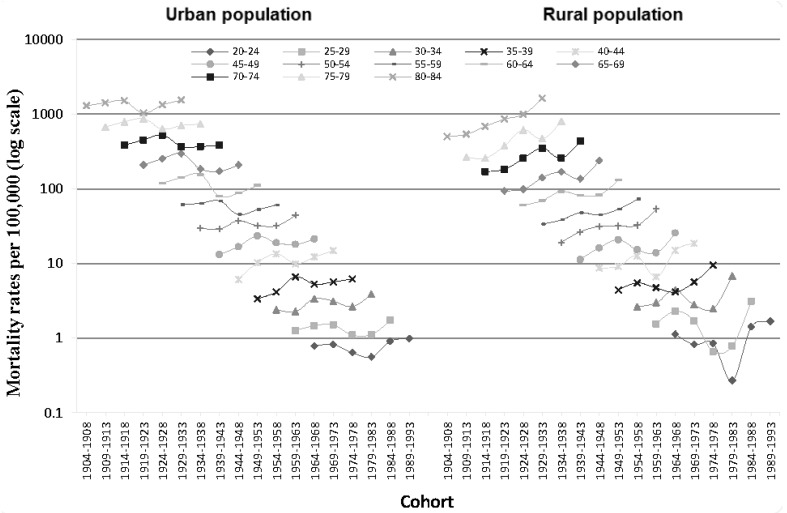
Cohort-specific IHD mortality rates in urban and rural China, by age group.

Figure 4 shows cohort-specific IHD mortality rates by age group. The influence of cohort on mortality differed by age group. While mortality among those aged 50–54 and 80–84 in rural areas was found to increase steadily with each successive cohort, there was no apparent trend in any other population group.

### 3.2. Statistical Analysis

Table 1 shows the results of each of the seven models fitted. The goodness-of-fit measures show that the full three-factor model accounting for age, period and cohort effects simultaneously provided the best prediction of IHD mortality rates over time.

**Figure 5:**
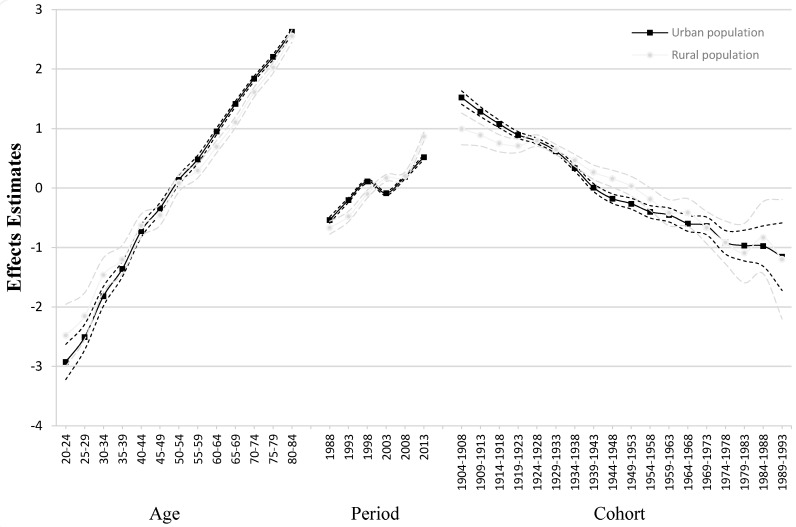
Age-period-cohort effects on IHD mortality in China.

Coefficients were then plotted to show the net effects of age, period, and cohort on overall IHD mortality trends, as shown in Figure 5. The corresponding results from the full APC model are displayed in Table 2. While the age effect most strongly influenced mortality rates, the period effect was relatively minor.

***Age effect.*** The age effect increased over time in an approximately log-linear fashion in urban and rural populations. While this effect was stronger in the latter, particularly among individuals aged under 44 years, it was more pronounced among those over 44 in urban areas. Overall, the influence of age on mortality increased faster in the urban population.

***Period effect.*** The influence of period effects increased in urban and rural populations throughout the study period. While the period effect was more pronounced during 1988–1998 in the urban population, period had a greater influence on mortality in the rural population from 2003 onwards. Overall the period effect followed a V-shaped trend during 1998–2008—although a continuous rising trend was seen in the rural population moderated from 2003 to 2008.

***Cohort effect.*** The influence of cohort on mortality decreased over time in both urban and rural populations. There were three increases in the birth cohort effect in the rural population born from 1919–1923 to 1924–1928, 1959–1963 to 1964–1968, and 1979–1983 to 1984–1988. The cohort effect was generally weaker in urban population among individuals born from 1929–1933 onwards and its influence on mortality was found to decrease over time—although, notably, this trend moderated among those born from 1974–1988.

## 4. Discussion

According to the Report on Cardiovascular Diseases in China (2013), the average annual growth rate in IHD mortality from 2004 to 2012 was 5.05%—a trend which is expected to continue over the next decade. Although previous studies have applied APC analysis to investigate the decline in IHD mortality over recent decades in developed countries [17,18,19,20], studies to examine the influence of age, period, and cohort effects on secular trends in IHD mortality in China have been scarce. The current study can provide insight into possible population-level risk factors driving the rapid rise in IHD mortality, and signpost avenues for future studies or public health intervention strategies.

Our findings regarding age effects on IHD mortality in both the urban and rural populations are in line with those from previous studies conducted in other countries [17,19,22], which indicate that IHD mortality is highly age-dependent. Although aging is a non-modifiable risk factor for IHD, it is accompanied by declines in health and physical functioning, and, as such, is positively associated with modifiable risk factors such as elevated blood pressure, diabetes mellitus, and hypercholesterolemia [31].

Population aging in China has gathered pace owing to the sharp decline in fertility and rising life expectancy. The number of people aged 65 and over increased from 49.28 million (or 4.91% of the population) in 1982 to 88.27 million (7.10%) in 2000. By 2010, this number rose to 118.93 million (8.92%, and 10.6% in rural areas) [32,33]. One previous study concluded that population aging is the dominant driver behind the increase in IHD mortality and resulted in a 27% rise mortality in Beijing from 1990 to 2010 [34]. Another predicted that coronary heart disease mortality will be 64% higher (resulting in 3.4 million additional deaths) in the decade 2020–2029 than in 2000–2009 solely owing to population growth and aging [23]. Our findings, together with those of previous studies, highlight the extent to which population aging has exacerbated the IHD disease burden in China.

Although similar age-specific patterns in IHD mortality were identified in urban and rural populations, it is notable that the age effect increased faster in the urban population. Furthermore, while the age effect was stronger in the rural population aged under 45 years, this effect was reversed among those over 45 years. China has become one of the countries with the severest urban air pollution problem in recent years [35]. One study conducted in 74 leading Chinese cities concluded that PM_2.5_ (particle less than 2.5 mm in aerodynamic diameter) was associated with 35% of cardiovascular mortality in 2013, which accounted for 47% of all PM_2.5_ related death of that year [36]. While many leading cities in China have become highly air polluted regions, however, the concentration of main air pollutants was somewhat lower in rural areas. This fact might relate to the slight difference in the changing pattern of age effects between urban and rural populations.

There was a falling trend in the influence of the cohort effect on IHD mortality for both urban and rural populations from 1904 to 1993—as has also been reported in Spain, Hungary, and South Korea [17,19,22]. Moreover, a recent study investigating secular trends in breast cancer mortality in China, South Korea, Japan, and United States employing APC analysis found that all countries experienced a similar general decline in the cohort effect over time despite differences in mortality trends [37]. This phenomenon may highlight that the Chinese population has substantially benefited from improvements in nutrition, living conditions, and availability of healthcare through much of the last century—resulting in a weakening of the cohort effect.

This stands in contrast, however, with recent population-level rising exposure to other modifiable risk factors such as, diabetes, cholesterolemia, and obesity. This trend may have the potential to strengthen the cohort effect—a point of concern raised by Wang et al. in interpreting a recent APC analysis [37,38]. Nevertheless, this is still reasonable, given that the effects of increases in these risk factors might have been offset by other driving forces in the opposite direction. Besides the successive overall improvements in nutrition, living conditions, and healthcare across birth cohorts in China in the last century, recent declines in some important risk factors of IHD might also have played a role. For example, while rates of smoking, a major modifiable lifestyle risk factor for IHD, remain relatively high, the recent decrease in smoking prevalence among Chinese men in most age groups, especially in younger age groups [39,40], may also have contributed to the decline in cohort effects on IHD mortality. However, caution should also be taken when interpreting the strength of the cohort effect in younger generations as their later-life risk of IHD could not be compared with that of previous generations. Furthermore, cumulative lifetime exposure to these other lifestyle risk factors may not yet be sufficient among younger generations to have influenced IHD mortality rates.

Period effects primarily result from changes in environmental determinants of mortality over time, which influence all age groups equally. Despite minor differences, our results showed that the period effect strengthened over time in both urban and rural populations. Although the magnitude of the age and cohort effects were comparatively larger, the period effect also represented an important factor influencing IHD mortality trends in China given its close association with trends in crude IHD mortality in urban and rural both populations. Similar results have been reported from studies using APC analysis to investigate secular trends in other chronic diseases in China [37,41]. One factor driving the increasing influence of the period effect on IHD mortality may be rising levels of air pollution, identified as the most significant environmental risk factor globally [42] and a major determinant of IHD morbidity and mortality [43] in China over recent decades. It is estimated that 9.4% of IHD mortality worldwide is attributable to exposure to anthropogenic PM_2.5_ alone [44], and recent studies have reported significant associations between ambient PM_2.5_ and PM_10_ (particle less than 10 mm in aerodynamic diameter) concentrations and IHD mortality among Chinese citizens [45,46,47]. Conversely, the sharp decrease in the strength of the period effect in the urban population from 1998 to 2003 may have been the result of changes in reporting following the move from ICD-9 to ICD-10 codes in 2002. The same pattern was not observed in rural populations over the same period, however. After adjustment of the data in ICD-9 format collected prior to 2002, the abnormal V-shape trend of period effects on urban IHD mortality disappeared.

The primary limitation of this study is that its results cannot describe the causal mechanisms through which age, period, and cohort influence mortality. Instead, APC analysis provides an exploratory tool to describe population-level patterns in mortality. By extension, to avoid succumbing to the ecological fallacy, it should be noted that our findings based on population-level data do not necessarily hold for individuals. Further analyses employing individual-level data are therefore required to identify these causal mechanisms. Another important limitation is the ICD change implemented with ICD-10 in 2002, which may have affected the mortality trends. However, the sensitivity analysis based on adjusted data showed that the impact of change in ICD on the APC model results was small, except for the trend of period effects in urban population. Thus, our interpretation of the results and the main conclusions were not influenced by the change in ICD.

## 5. Conclusions

Our findings indicate that China’s rapid population aging in recent years has played a critical role in driving the rising trend in IHD mortality in both urban and rural populations. Period effects, which may reflect the population-wide increase in exposure to environmental risk factors such as outdoor air pollution, also represent a major factor influencing the trend in mortality. Although lifestyle risk factors resulting from changing diets and physical inactivity are highly prevalent among younger generations, their long-term effects may not yet have manifested in a notable cohort effect on IHD mortality. More evidence, however, is required to identify the factors driving the upward trend in IHD mortality in the world’s most populous country and elucidate their causal mechanisms.

Although population aging is inevitable, there is scope for policymakers in China and other countries confronting the epidemiologic transition to reduce the disease burden and mortality rates due to IHD. Possible avenues for intervention could include improving social protection and healthcare provision for elderly populations, extending public health insurance to cover the spending of primary healthcare in the community, where most management of IHD risk factors takes place, undertaking measures to limit exposure to air pollution and other environmental risk factors, and implementing public education programs to reduce lifestyle risk factors.

## Figures and Tables

**Table 1:** Goodness-of-fit statistics for age-period-cohort log linear models of China ischemic heart disease (IHD) mortality rates between 1987 and 2013.

	Age	Period	Cohort	Age-Period	Age-Cohort	Period-Cohort	Age-Period-Cohort
Urban population							
Deviance	19,896.83	2,899,129.19	648,370.43	7322.31	14,118.80	3152.12	1653.67
AIC	265.72	37,178.78	9349.67	104.64	192.08	51.30	32.37
BIC	19,613.65	2,898,816	648,109	7060.91	13,909.68	2912.50	1461.98
DOF	65	72	60	60	48	55	44
Rural population							
Deviance	230,787.78	5,216,118.22	2,004,389.54	28,468.26	47,406.37	23,521.58	14,191.24
AIC	2970.23	66,884.54	25,708.84	376.51	619.62	313.22	193.88
BIC	230,504.60	5,215,805	2,004,128	28,206.85	47,197.24	23,281.96	13,999.55
DOF	65	72	60	60	48	55	44

AIC = Akaike’s information criterion; BIC = Bayesian information criterion; DOF = Degree of freedom. The smaller the AIC and BIC, the better the model fit.

**Table 2:** Intrinsic estimates for IHD mortality rates in urban and rural Chinese population.

	Urban Population		Rural Population	
	Coeff.	S.E.	Coeff.	S.E.
Intercept	−7.97 *	0.02	−8.19 *	0.04
Age (Year)				
20–24	−2.92 *	0.15	−2.48 *	0.27
25–29	−2.51 *	0.11	−2.15 *	0.20
30–34	−1.82 *	0.08	−1.46 *	0.15
35–39	−1.36 *	0.07	−1.20 *	0.12
40–44	−0.72 *	0.05	−0.63 *	0.10
45–49	−0.34 *	0.05	−0.45	0.09
50–54	0.14 *	0.04	0.08 *	0.07
55–59	0.48 *	0.03	0.29 *	0.06
60–64	0.95 *	0.03	0.70 *	0.05
65–69	1.42 *	0.02	1.11 *	0.05
70–74	1.84 *	0.02	1.62 *	0.04
75–79	2.21 *	0.02	2.02 *	0.05
80–84	2.63 *	0.03	2.56 *	0.05
Period (Year)				
1988	−0.54 *	0.03	−0.67 *	0.06
1993	−0.20 *	0.02	−0.48 *	0.04
1998	0.11 *	0.02	−0.10 *	0.03
2003	−0.09 *	0.02	0.17 *	0.03
2008	0.19 *	0.02	0.21 *	0.03
2013	0.52 *	0.02	0.87 *	0.04
Cohort (Year)				
1904–1908	1.52 *	0.06	0.99 *	0.14
1909–1913	1.28 *	0.04	0.89 *	0.09
1914–1918	1.08 *	0.03	0.75 *	0.07
1919–1923	0.89 *	0.03	0.71 *	0.06
1924–1928	0.79	0.02	0.80 *	0.05
1929–1933	0.62 *	0.02	0.65 *	0.04
1934–1938	0.34 *	0.03	0.47	0.05
1939–1943	0.01 *	0.03	0.27	0.06
1944–1948	−0.18 *	0.04	0.16 *	0.07
1949–1953	−0.26 *	0.05	0.03 *	0.08
1954–1958	−0.40 *	0.05	−0.19 *	0.10
1959–1963	−0.45 *	0.06	−0.41 *	0.11
1964–1968	−0.60 *	0.07	−0.41 *	0.12
1969–1973	−0.64 *	0.08	−0.67 *	0.14
1974–1978	−0.91 *	0.10	−0.92 *	0.18
1979–1983	−0.97 *	0.13	−1.09 *	0.26
1984–1988	−0.98 *	0.17	−0.83 *	0.31
1989–1993	−1.15 *	0.29	−1.20 *	0.51

* *p* < 0.05; exponentiated coefficients interpreted as odds ratios; Coeff. = coefficient; S.E. = standard error of the coefficients.

## Supplementary Materials

The following are available online at www.mdpi.com/1660-4601/14/1/50/s1, Figure S1: Adjusted ischemic heart disease (IHD) mortality rates trend in urban and rural China, 1987–2013, Figure S2: Age-period-cohort effects on IHD mortality in China (adjusted results), Figure S3: (a) Comparability between adjusted and unadjusted results of age effects on IHD mortality; (b) Comparability between adjusted and unadjusted results of period effects on IHD mortality; (c) Comparability between adjusted and unadjusted results of cohort effects on IHD mortality, Table S1: Intrinsic estimates for adjusted ischemic heart disease (IHD) mortality rates in urban and rural Chinese population.
