# Supplementary material for: The Effects of Age, Period, and Cohort on Mortality from Ischemic Heart Disease in China
Source: Int J Environ Res Public Health. 2017 Jan 7;14(1):50. doi: 10.3390/ijerph14010050 (PMC5295301; doi:10.3390/ijerph14010050)
Supplement: Supplementary file 1 [file ijerph-14-00050-s001.pdf]

Supplementary Materials: The Effects of Age, Period and Cohort on Mortality from Ischemic Heart Disease in China

Jie Chang, Boyang Li, Jingjing Li and Yang Sun

Table S1. Intrinsic estimates for adjusted ischemic heart disease (IHD) mortality rates in urban and rural Chinese population.

	Urban Population			Rural Population		
	**Coeff.**	**exp(Coeff.)**	**SE**	**Coeff.**	**exp(Coeff.)**	**SE**
**Intercept**	−8.15 *	0.00 *	0.02	−8.23 *	0.00 *	0.04
**Age (Year)**						
20–24	−2.94 *	0.05 *	0.15	−2.49 *	0.08 *	0.27
25–29	−2.53 *	0.08 *	0.11	−2.17 *	0.11 *	0.20
30–34	−1.81 *	0.16 *	0.08	−1.46 *	0.23 *	0.15
35–39	−1.36 *	0.26 *	0.07	−1.20 *	0.30 *	0.13
40–44	−0.72 *	0.49 *	0.05	−0.63 *	0.53 *	0.10
45–49	−0.34 *	0.71 *	0.05	−0.45 *	0.64 *	0.09
50–54	0.14 *	1.16 *	0.04	0.09	1.10	0.08
55–59	0.48 *	1.61 *	0.03	0.30 *	1.35 *	0.06
60–64	0.94 *	2.57 *	0.03	0.70 *	2.01 *	0.05
65–69	1.42 *	4.13 *	0.02	1.11 *	3.04 *	0.05
70–74	1.85 *	6.35 *	0.02	1.62 *	5.05 *	0.04
75–79	2.21 *	9.15 *	0.02	2.03 *	7.58 *	0.05
80–84	2.65 *	14.11 *	0.03	2.56 *	12.89 *	0.05
**Period (Year)**						
1988	−0.71 *	0.49 *	0.03	−0.71 *	0.49 *	0.06
1993	−0.38 *	0.69 *	0.02	−0.52 *	0.59 *	0.05
1998	−0.07 *	0.93 *	0.02	−0.15 *	0.86 *	0.04
2003	0.09 *	1.10 *	0.02	0.21 *	1.24 *	0.03
2008	0.37 *	1.44 *	0.02	0.25 *	1.29 *	0.03
2013	0.70 *	2.01 *	0.02	0.92 *	2.50 *	0.04
**Cohort (Year)**						
1904–1908	1.50 *	4.46 *	0.06	1.00 *	2.71 *	0.14
1909–1913	1.26 *	3.54 *	0.04	0.89 *	2.44 *	0.10
1914–1918	1.06 *	2.89 *	0.03	0.75 *	2.13 *	0.08
1919–1923	0.88 *	2.40 *	0.03	0.71 *	2.04 *	0.06
1924–1928	0.79 *	2.19 *	0.02	0.80 *	2.22 *	0.05
1929–1933	0.62 *	1.85 *	0.02	0.65 *	1.91 *	0.04
1934–1938	0.33 *	1.40 *	0.03	0.46 *	1.59 *	0.05
1939–1943	0.01	1.01	0.03	0.26 *	1.30 *	0.06
1944–1948	−0.17 *	0.84 *	0.04	0.15 *	1.16 *	0.07
1949–1953	−0.25 *	0.78 *	0.05	0.02	1.03	0.08
1954–1958	−0.39 *	0.68 *	0.05	−0.19 *	0.83 *	0.10
1959–1963	−0.45 *	0.64 *	0.06	−0.42 *	0.66 *	0.11
1964–1968	−0.59 *	0.55 *	0.07	−0.42 *	0.66 *	0.12
1969–1973	−0.63 *	0.53 *	0.07	−0.67 *	0.51 *	0.14
1974–1978	−0.91 *	0.40 *	0.10	−0.91 *	0.40 *	0.18
1979–1983	−0.96 *	0.38 *	0.12	−1.09 *	0.34 *	0.25
1984–1988	−0.95 *	0.39 *	0.16	−0.82 *	0.44 *	0.31
1989–1993	−1.13 *	0.32 *	0.27	−1.19 *	0.31 *	0.51
**Deviance**		1381.46			13771.45	
**DOF**		44			44	
**AIC**		28.6994			188.4577	
**BIC**		1189.771			13579.76	

* $p < 0.05$; exponentiated coefficients interpreted as odds ratios. Coeff.: coefficient; exp(Coeff.): relative risk; SE: standard error of the coefficients; AIC: Akaike's information criterion; BIC: Bayesian information criterion; DOF: degree of freedom. The smaller the AIC and BIC, the better the model fit.

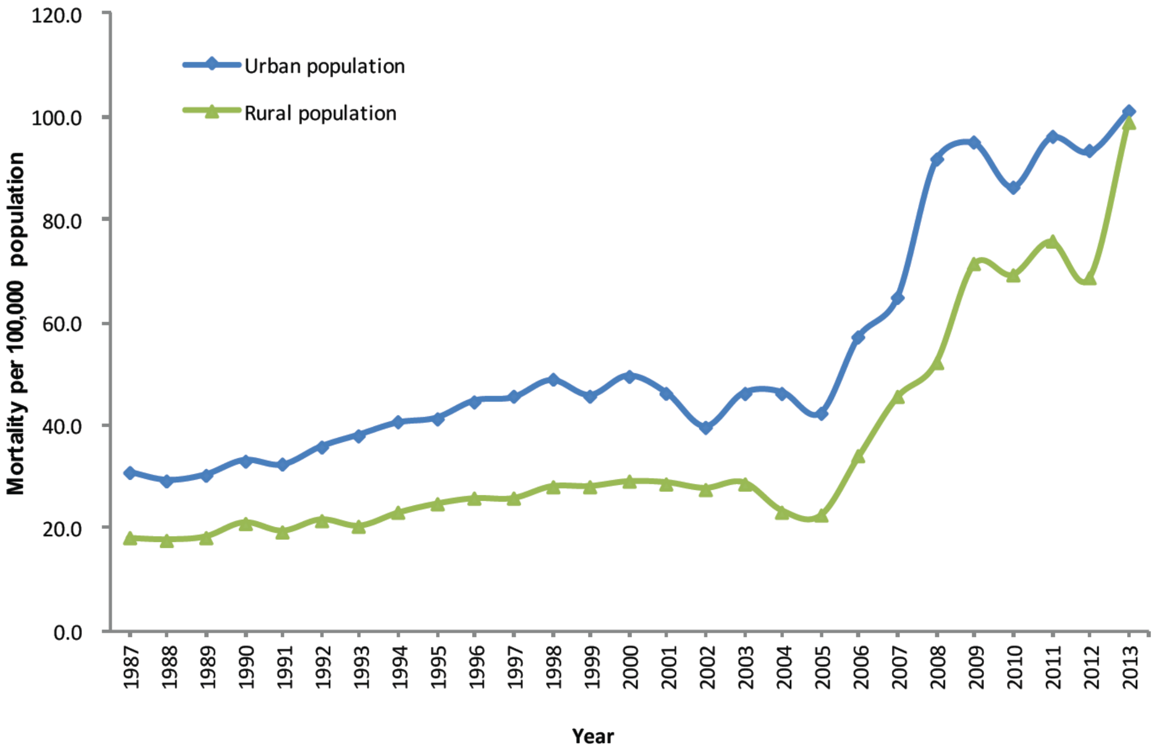

Figure S1. Adjusted ischemic heart disease (IHD) mortality rates trend in urban and rural China, 1987–2013.

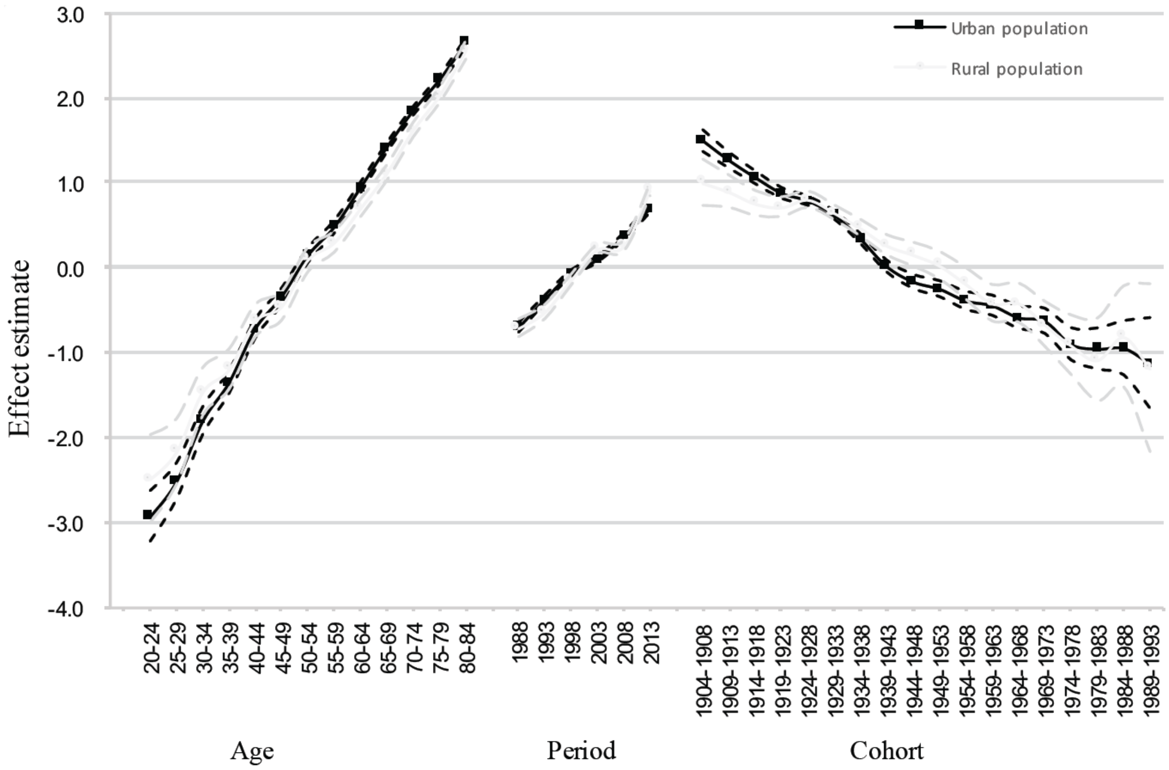

Figure S2. Age-period-cohort effects on IHD mortality in China (adjusted results).

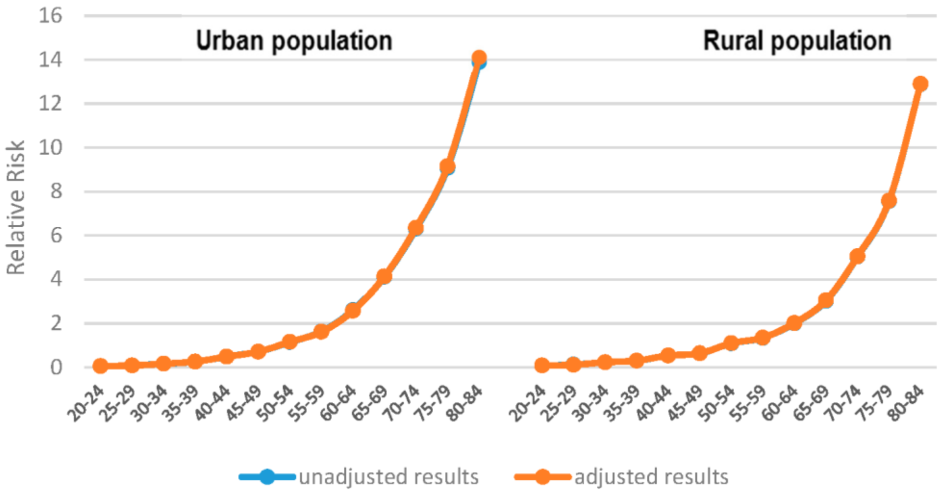

(A)

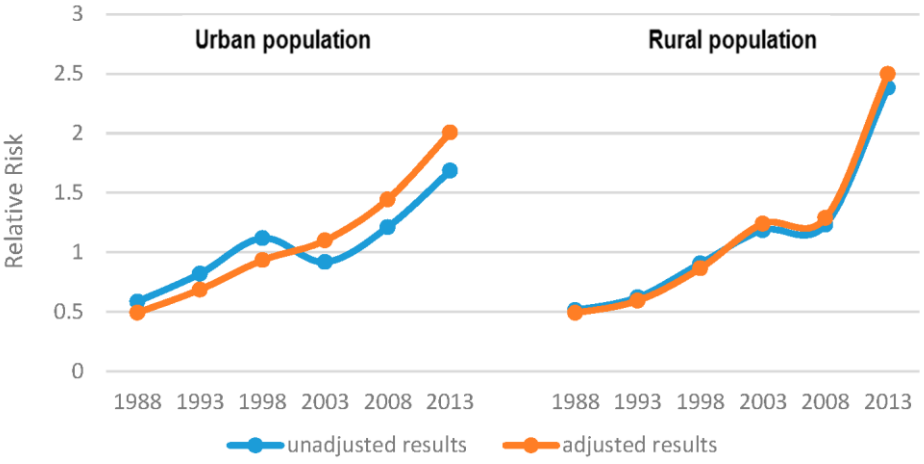

(B)

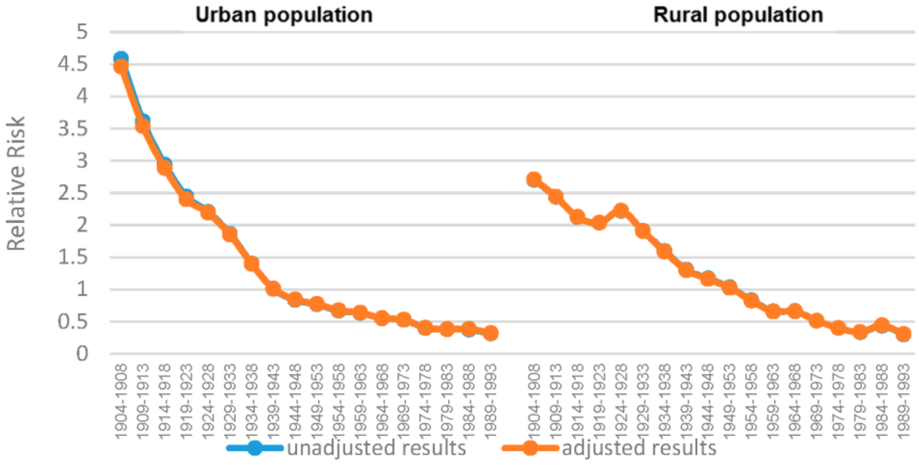

(C)

Figure S3. (**A**). Comparability between adjusted and unadjusted results of age effects on IHD mortality. (**B**) Comparability between adjusted and unadjusted results of period effects on IHD mortality. (**C**) Comparability between adjusted and unadjusted results of cohort effects on IHD mortality.
